# Urine 5MedC, a Marker of DNA Methylation, in the Progression of Chronic Kidney Disease

**DOI:** 10.1155/2019/5432453

**Published:** 2019-07-01

**Authors:** Akifumi Onishi, Hitoshi Sugiyama, Masashi Kitagawa, Toshio Yamanari, Keiko Tanaka, Ayu Ogawa-Akiyama, Yuzuki Kano, Koki Mise, Katsuyuki Tanabe, Hiroshi Morinaga, Masaru Kinomura, Haruhito A. Uchida, Jun Wada

**Affiliations:** ^1^Department of Nephrology, Rheumatology, Endocrinology and Metabolism, Okayama University Graduate School of Medicine, Dentistry and Pharmaceutical Sciences, Okayama, Japan; ^2^Department of Human Resource Development of Dialysis Therapy for Kidney Disease, Okayama University Graduate School of Medicine, Dentistry and Pharmaceutical Sciences, Okayama, Japan; ^3^Department of Molecular Life Sciences, Tokai University School of Medicine, Kanagawa, Japan; ^4^Division of Medical Informatics, Okayama University Hospital, Okayama University Graduate School of Medicine, Dentistry and Pharmaceutical Sciences, Okayama, Japan; ^5^Department of Chronic Kidney Disease and Cardiovascular Disease, Okayama University Graduate School of Medicine, Dentistry and Pharmaceutical Sciences, Okayama, Japan

## Abstract

**Background:**

Alterations in DNA methylation may be involved in disease progression in patients with chronic kidney disease (CKD). Recent studies have suggested that 5-methyl-2′-deoxycytidine (5MedC) may be a marker of hypermethylation of DNA. Currently, there is no information available regarding the urine levels of 5MedC and its association with the progression of CKD.

**Method:**

We examined the urine levels of 5MedC in spot urine samples from 308 patients with CKD (median age: 56 years, male: 53.2%, and glomerulonephritis: 51.0%) using a competitive enzyme-linked immunosorbent assay and investigated the relationships among urine 5MedC, urine albumin, urine *α*1-microglobulin (*α*1MG), and the laboratory parameters associated with CKD. The patients were followed for three years to evaluate renal endpoints in a prospective manner.

**Results:**

The urine 5MedC level was significantly increased in the later stages of CKD compared to the early to middle stages of CKD. In multiple logistic regression models, urine 5MedC was significantly associated with the prediction of later CKD stages. Urine 5MedC (median value, 65.9 *μ*mol/gCr) was significantly able to predict a 30% decline in the estimated GFR or a development of end-stage renal disease when combined with macroalbuminuria or an increased level of urine *α*1MG (median value, 5.7 mg/gCr).

**Conclusion:**

The present data demonstrate that the urine 5MedC level is associated with a reduced renal function and can serve as a novel and potent biomarker for predicting the renal outcome in CKD patients. Further studies will be necessary to elucidate the role of urine DNA methylation in the progression of CKD.

## 1. Introduction

Epigenetic changes are stable and heritable but reversible modifications, including DNA methylation, posttranscriptional modifications of histone, and remodeling of chromatin [1, 2]. Among them, DNA methylation is a crucial epigenetic alteration observed in eukaryotic organisms and has been shown to be associated with many biological and cellular processes, such as embryonic development, transcription, structure of chromatin, and stability of chromosome [3, 4]. Recently, several human diseases have been reported to be associated with abnormal DNA methylation [4, 5].

Chronic kidney disease (CKD) is a significant concern given the increasing number of such patients throughout the entire world [6]. CKD is characterized by either or both a glomerular filtration rate (GFR) less than 60 mL/min/1.73 m^2^ and signs of kidney injury of at least 3 months' duration [6, 7]. A reduced estimated GFR (eGFR) and severe degree of albuminuria independently predict end-stage renal disease and mortality in CKD patients [8]. There is an urgent need to identify novel biomarkers in patients with CKD in order to better detect those at high risk of a rapid decline in the renal function so that effective therapies can be used to inhibit the disease progression [9].

A recent large-scale genome-wide evaluation of DNA methylation showed that DNA hypomethylation and hypermethylation were present at different loci in patients with CKD [10]. DNA hypermethylation in the peripheral blood may be linked to inflammation possibly associated with bacterial infections in CKD patients with incident dialysis [11].

5-Methyl-2′-deoxycytidine (5MedC) (PubChem CID: 440055) is a product of the base excision repair (BER) and nucleotide excision repair (NER) pathways of active DNA methylation. 5MedC was detected in the urine of healthy individuals as well as in those with certain diseases via several methods [12–16]; however, little is known about its levels in the urine of CKD patients or its association with progression of disease in such patients.

We therefore determined the urine levels of 5MedC and its association with progression and renal outcome in patients with CKD.

## 2. Methods

### 2.1. Study Design

The study subjects were outpatients who had visited the Division of Nephrology in Okayama University Hospital between February 2009 and February 2012. All patients were diagnosed with CKD in accordance with the eGFR and the presence of kidney damage, as characterized by the National Kidney Foundation K/DOQI Guideline [6]. The eGFR was calculated as described previously [17]. Hypertension was defined as described previously [18, 19]. The mean blood pressure (MBP) was calculated as diastolic blood pressure + (systolic blood pressure − diastolic pressure)/3.

All procedures in the current study were performed according to national and institutional ethical guidelines of human studies and guidelines in the Declaration of Helsinki. The ethics committee of Okayama University Graduate School of Medicine, Dentistry and Pharmaceutical Sciences approved the study (KEN1607-010). All subjects gave written informed consent.

A prospective and longitudinal study was performed to investigate the relationship among urine 5MedC levels, clinical parameters, and the renal outcome in CKD patients. The patients who participated in this study were recruited between February 2009 and February 2012. Patients were followed for up to 3 years, but those who were followed for less than 3 months (*n* = 51) or who started renal replacement therapy within 3 months of the participation (*n* = 5) were excluded. As a result, a total of 308 patients were included in the analysis. Of these patients, 199 patients (male 107, female 92) were overlapped with the previous study [19]. In accordance with the established protocol, we excluded any patients with established atherosclerotic complications (congestive heart failure, coronary artery disease, or peripheral vascular disease) [19]. Patients with infection, acute kidney injury, cancer, and Alzheimer's disease at entry were also excluded.

### 2.2. Laboratory Measurement of Urine Biomarkers

Spot urine samples were collected from patients in the morning, as described previously [19]. The urine 5MedC levels were measured using a Global DNA Methylation Enzyme-Linked Immunosorbent Assay (ELISA) Kit (Cell Biolabs Inc., San Diego, CA, USA), which was a competitive enzyme immunoassay developed for the rapid quantitation and detection of 5MedC in urine directly. The quantity of 5MedC in an unknown sample is calculated by comparing its absorbance with that of a known 5MedC standard curve. The kit has a 5MedC detection sensitivity range of 150 nM to 10 *μ*M. The concentration of 8-hydroxy-2′-deoxyguanosine (8-OHdG) in urine samples was also determined using an ELISA kit (R&D Systems, Minneapolis, MN, USA) as previously described [20, 21].

The median duration of storage between collection of urine and measurement of biomarkers was 48 months (interquartile range, 34-49 months). The urine levels of albumin, total protein, creatinine (Cr), and alpha1-microglobulin (*α*1MG) were measured by standard methods and used to calculate the urine albumin-to-Cr ratio (urinary albumin excretion (UAE)), urine *α*1MG-to-Cr ratio, and urine 5MedC-to-Cr ratio.

### 2.3. Data Collection

Each subject's age, gender, cause of CKD, complication of diabetes mellitus, medication with antihypertensive drugs (angiotensin receptor blocker (ARB), angiotensin-converting enzyme inhibitor (ACEI), calcium channel blocker (CCB)), mean blood pressure (MBP), and other clinical laboratory data were collected. The serum creatinine concentration was measured by the enzymatic colorimetric method using an automated analyzer (JCA-BM8040; JEOL, Tokyo, Japan), as described previously [19].

### 2.4. Evaluation of Outcome

The primary outcome was CKD progression, defined as a composite endpoint of incident end-stage renal disease (recipient of maintenance dialysis or renal transplant) or a 30% decline in the eGFR [22, 23]. Patients were prospectively followed for a median of 36 months (interquartile range, 26–37 months). They were followed by a review of the medical record at least twice a year until December 31, 2014. Loss to follow-up and death were considered censoring events, as described previously [19].

### 2.5. Statistical Analyses

All values were indicated as the median (interquartile range) or number (percentage). Differences between groups were compared using Wilcoxon's test, a *t*-test, or log-rank test. Kaplan-Meier analyses were applied to assess the effect of urine 5MedC levels on the renal endpoint using a generalized Wilcoxon test [19, 21]. A multiple regression analysis was used to evaluate the predictors of a low eGFR using the odds ratio (OR) or adjusted OR after adjusting for relevant factors [18, 19, 21]. A *P* value of <0.05 was considered to be statistically significant. The SPSS version 20 software package (SPSS Inc., Chicago, IL, USA) and JMP version 11 program (SAS Institute Inc., Cary, NC, USA) were utilized to perform the statistical analyses.

## 3. Results

### 3.1. Urine 5MedC Levels and CKD Stages

**Figure 1:**
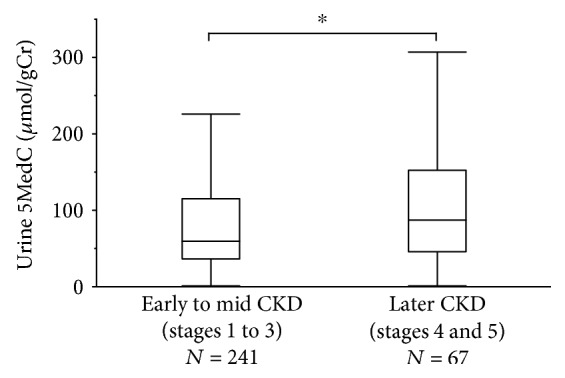
Urine 5MedC and CKD stages. Box and line plots showing the levels of urine 5MedC (*μ*mol/gCr) according to the CKD stages (early to middle stages 1 to 3 or later stages 4 and 5) based on the estimated glomerular filtration rate. The boxes denote the medians and 25th and 75th percentiles. The lines mark the 5th and 95th percentiles. Wilcoxon's test. CKD: chronic kidney disease; 5MedC: 5-methyl-2′-deoxycytidine.

The baseline profiles of the study subjects are summarized in accordance with the early to middle (stages 1 to 3) and later (stages 4 and 5) stages of CKD (Table 1). This study included 308 patients (male, *n* = 164; female, *n* = 144) with a median age of 56 (37-67) years. The background cause of CKD in more than half of the cases was chronic glomerulonephritis (51.0%). This distribution of patients with chronic glomerulonephritis was similar to that in other nephrology divisions reported in the Japan Renal Biopsy Registry [24]. Significant increases in the levels of albuminuria, urine *α*1MG, and MBP as well as significant decreases in hemoglobin were recognized, resembling those reported in other cohorts of CKD [6, 25, 26]. The median values of urine 5MedC were 59.7 and 88.3 *μ*mol/gCr in the early to middle and later CKD stages, respectively (Figure 1). The concentrations of urine 5MedC were significantly increased in later stages of CKD, suggesting its association with disease progression (Figure 1).

### 3.2. A Multivariate Analysis to Determine a Low eGFR (Less than 30 mL/min/1.73 m^2^) in Patients with CKD

Next, separate multiple logistic regression analyses to determine a low eGFR (<30 mL/min/1.73 m^2^), which is equivalent to advanced CKD stages 4 and 5, were performed (Table 2). The urine 5MedC level alone was elucidated to be a significantly independent predictor of a low eGFR (model 1). After adjusting for gender and age as confounding parameters, the urine 5MedC level was still significant in model 2, which included albuminuria, and model 3, which further included u*α*1MG (Table 2). In the univariate analysis, there were no significant correlations between urine 5MedC and other parameters (Table S1). The urine 5MedC levels did not significantly differ when categorized according to the age, gender, cause of CKD, or complications (Table S2).

### 3.3. Urine 5MedC in Combination with Other Urine Proteins Significantly Predicts the Renal Survival

**Figure 2:**
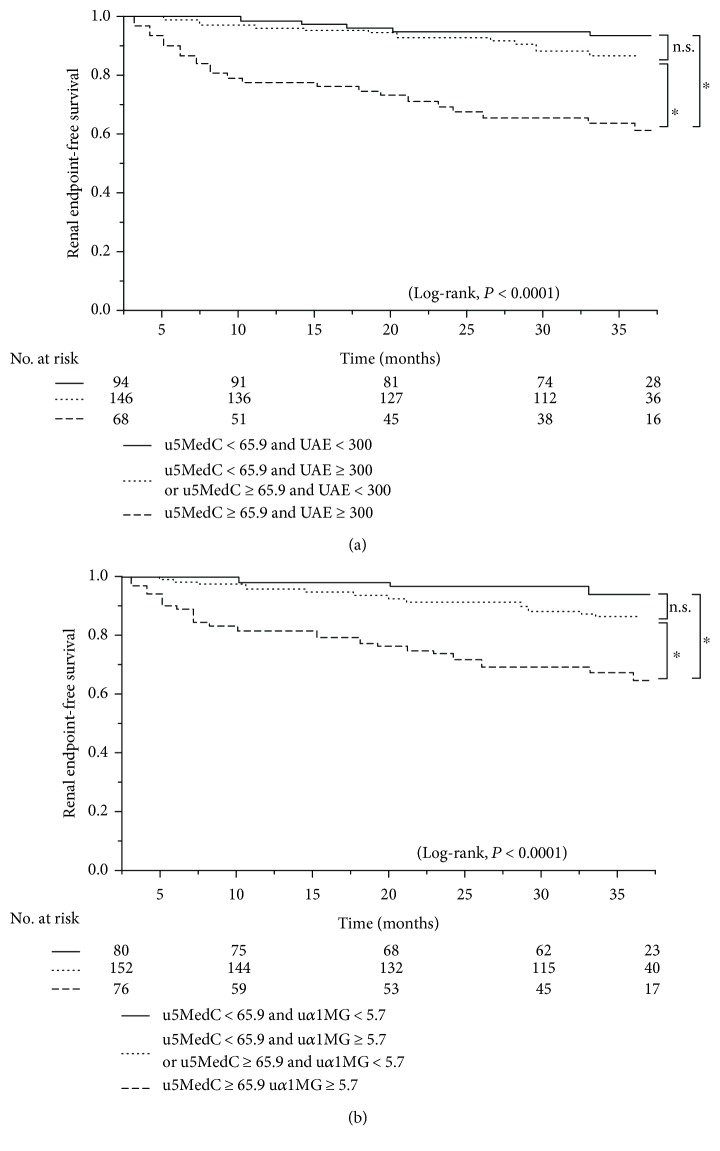
Urine 5MedC and CKD outcome. Kaplan-Meier curves showing the renal endpoint-free survival categorized by urine 5MedC (*μ*mol/gCr) and its combination with albuminuria (mg/gCr) (a) or urine *α*1MG (mg/gCr) (b). The combination of urine 5MedC with albuminuria (a) or urine *α*1MG (b) clearly separated the three-year renal endpoint-free survival of CKD patients. (a) u5MedC < 65.9 and UAE < 300, *n* = 94 (30.5%); u5MedC < 65.9 and UAE ≥ 300 or u5MedC ≥ 65.9 and UAE < 300, *n* = 146 (47.4%); and u5MedC ≥ 65.9 and UAE ≥ 300, *n* = 68 (22.1%). (b) u5MedC < 65.9 and u*α*1MG < 5.7, *n* = 80 (26.0%); u5MedC < 65.9 and u*α*1MG ≥ 5.7 or u5MedC ≥ 65.9 and u*α*1MG < 5.7, *n* = 152 (49.4%); u5MedC ≥ 65.9 and u*α*1MG ≥ 5.7, *n* = 76 (24.7%). ∗ indicates *P* < 0.0001, n.s. denotes not significant. Log-rank test. UAE: urinary albumin excretion; u*α*1MG: urinary alpha1-microglobulin; u5MedC: urinary 5-methyl-2′-deoxycytidine.

During the 36 months of follow-up, 46 patients exhibited a 30% decline in the eGFR (*n* = 24) or developed end-stage renal disease requiring renal replacement therapy (*n* = 22). There was a higher incidence of disease progression in patients with advanced CKD (stages 4 to 5) (33 of 67 patients) than in those with early to middle CKD (stages 1 to 3) (13 of 241 patients). The baseline levels of albuminuria (<300 mg/gCr or ≥300 mg/gCr) or urine *α*1MG (median value, 5.7 mg/gCr) were able to predict the renal endpoint-free survival (Figure S1), suggesting that the CKD cohort in this study was consistent with the relative risk prediction model of CKD [6, 25, 26]. Several studies have investigated the combination of biomarkers to better predict the renal prognosis [19, 26]. We further performed survival analyses using the level of urine 5MedC (median value, 65.9 *μ*mol/gCr) in combination with the level of urine albumin (<300 mg/gCr or ≥300 mg/gCr) or with that of urine *α*1MG (median value, 5.7 mg/gCr) in Kaplan-Meier survival curves (Figure 2). An increased urine 5MedC level in CKD patients did not significantly predict a worse renal outcome than a lower urine 5MedC level (Figure S1); however, a significant effect of an increased urine 5MedC level on predicting a poor renal survival when combined with macroalbuminuria (≥300 mg/gCr) or an increased urine *α*1MG level was observed (Figure 2).

### 3.4. Relationship between Urine 5MedC, a Marker of DNA Methylation, and Urine 8-OHdG, a Marker of Oxidized DNA

**Figure 3:**
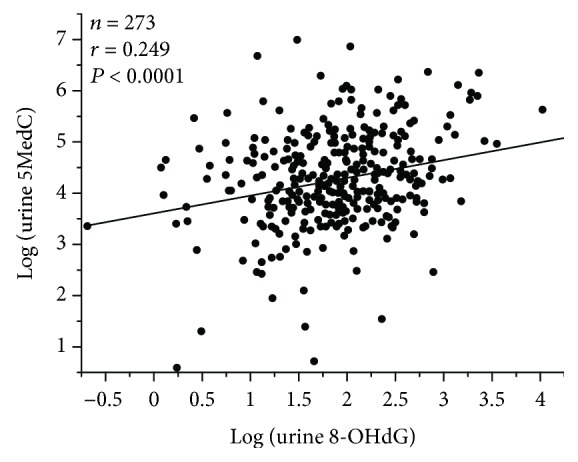
Relationship between levels of urine 5MedC and urine 8-OHdG. The level of urine 5MedC (*μ*mol/gCr) significantly correlates with the level of urine 8-OHdG (ng/mgCr) in patients with CKD (*n* = 273). *t*-test. 5MedC: 5-methyl-2′-deoxycytidine; 8-OHdG: 8-hydroxy-2′-deoxyguanosine.

We carried out a further analysis of the 273 patients with available data for urine 8-OHdG, a marker of oxidized DNA due to oxidative stress. We recognized a significant univariate correlation between 5MedC and 8-OHdG in the urine of CKD patients, suggesting an association between DNA methylation and oxidized DNA and thus a linkage between epigenetic and genetic alterations in such patients (Figure 3).

## 4. Discussion

In the genomic DNA of mammals, methylation of the C-5 position of cytosine is a key mechanism of epigenetic control that influences the gene expression, stability of genome, and differentiation of cells [27]. Abnormal methylation of several genes, either hypermethylation or hypomethylation, has been involved in various diseases, including cancer [2, 28, 29], diabetes [30], obesity [31], Alzheimer's disease [32], and schizophrenia [5]. The level of 5-methylcytidine (5MeC) is determined by the balance between DNA methylation and DNA demethylation processes. DNA methylation is catalyzed by DNA methyltransferases, with S-adenosylmethionine functioning as a donor of methyl. DNA methylation may be removed enzymatically by certain mechanisms including BER [33], NER, and hydrolysis [34–36].

In this study, we measured the urine level of 5MedC, a marker of repair products of DNA methylation, in patients with CKD and investigated the relationships between the urine 5MedC level and CKD progression and outcomes. Herein, we provide the evidence that (1) the urine 5MedC level was significantly increased in the later stages of CKD (i.e., eGFR less than 30 mL/min/1.73 m^2^) and was associated with a later CKD stage according to a multiple regression analysis even after adjusting for confounding parameters; furthermore, (2) while urine 5MedC alone did not significantly predict the renal outcome in CKD patients, a significant effect of urine 5MedC on predicting a poor renal outcome when combined with macroalbuminuria or an increased urine *α*1MG level was detected.

5MedC is a product of the BER and NER pathways of active DNA methylation. DNA repair products, including 5MedC, 5-hydroxymethylcytosine, 5-formylcytosine, and 5-carboxycytosine, are released into the blood and subsequently appear in the urine [37, 38]. Several techniques have been developed for the determination of 5MedC in human urine samples, such as immunochemical detection [13], ion-pair liquid chromatography (LC) [16], LC with mass spectrometry (LC-MS) [14], LC with tandem mass spectrometry (LC-MS/MS) [12], and high-performance LC with tandem mass spectrometry (HPLC-MS/MS) [15, 39].

Itoh et al. reported that an ELISA with specific monoclonal antibodies was able to detect 5MedC as the major immunoreactive nucleoside in the urine of a healthy human and increased concentrations of urine 5MedC were observed in leukemic patients with active diseases [13]. The mean levels of urine 5MedC in healthy subjects were 0.90 ± 0.43 nmol/*μ*molCr in that analysis. The generation of 5MedC may be caused by the active excision repair of DNA in human cells. Heavily methylated DNA of leukemic cells may be the origin of increased 5MedC in the urine of leukemic patients. Zambonin et al. then applied a simple reversed-phase LC technique to determine the urine 5MedC levels normalized by urine creatinine excretion in healthy individuals and patients with leukemia [16]. Lee et al. further analyzed the urine levels of oxidized nucleosides using LC with electrospray mass spectrometry and found that the urine 5MedC levels did not significantly change, but those of 8-OHdG were significantly elevated in patients with Alzheimer's disease compared to healthy subjects [14]. The mean urine level of 5MedC was 0.262 ± 0.156 nmol/*μ*molCr in that study. Based on these previous findings, we initially excluded patients with cancer and Alzheimer's disease from the present study.

Hu et al. measured the level of urine 5MeC and 5MedC by LC-MS/MS with isotope dilution in healthy males and found that the concentration of urine 5MeC was significantly correlated with those of methylated purines and lesions of oxidized DNA, including 8-oxo-7,8-dihydro-2′-deoxyguanosine (8-oxodG) [12]. The mean urine level of 5MedC was 7.04 ± 7.2 ng/mgCr in that report. The level of urine 5MedC, however, did not correlate with any methylated or oxidized lesions in healthy male subjects in that study. Pan et al. investigated the levels of urine 5MedC and 5-hydroxymethyl-2′-deoxycytidine (5hMedC) by HPLC-MS/MS in subfertile men and showed their associations with phthalate metabolites (environmental chemicals) and semen parameters (healthy outcomes), suggesting that these are promising biomarkers for use in epidemiological studies [15, 39]. In addition to urine samples of humans, other researchers have attempted to determine the 5MedC level in DNA obtained from human peripheral blood [40] or human lung cancer tissue [41] by LC-MS/MS as well as in DNA obtained from cultured Hela cells by HPLC-ultraviolet detection [42] and from newborn cord blood samples by HPLC-MS/MS [43].

Recent reports have identified roles of environmental [6], genetic [44, 45], and epigenetic factors [46, 47] in the progression of CKD. Epigenetic risk factors for CKD have only recently been investigated [10], and the DNA methylation profile in the blood might be associated with a rapid decline in the renal function [48]. In the present study, the group exhibiting both higher levels of urine 5MedC and albuminuria had a worse renal survival than the group exhibiting lower levels of both (Figure 2). Whether albuminuria induces epigenetic changes, including DNA methylation, and thus an increase in urine 5MedC in resident kidney cells is largely unknown. The expression of Krüppel-like factor 4, which can reprogram somatic cells into induced pluripotent stem cells, reduced DNA methylation at the nephrin promoter, which may lead to protection against albuminuria [49]. The hypomethylation of *aldo-keto reductase family 1 member B1* and *tissue inhibitor of metalloproteinase 2* genes in association with albuminuria has been reported in subjects with early stages of diabetic nephropathy [50], although we did not recognize a significant correlation between urine 5MedC and albuminuria levels in the univariate analysis in our cohort (Table S1).

We found in the present study that urine 5MedC levels were significantly increased in the later stages of CKD (stages 4 and 5, i.e., eGFR less than 30 mL/min/1.73 m^2^) (Figure 1), when uremic toxins may be detected in both the urine and serum of such patients. In recent reports, uremia was shown to induce alterations in DNA methylation in differentiating monocytes in patients with CKD [51]. The expression of the antiaging and renoprotective gene *klotho* is known to be suppressed under conditions of uremia [18]. The protein-bound uremic toxins can increase the DNA methyltransferase and DNA methylation, thereby leading to the suppression of the *klotho* expression in the uremic milieu [52]. Therefore, certain uremic toxins might alter the global DNA methylation and the expression of urine 5MedC in CKD patients. In rodent models, hypermethylation of certain genes is involved in the activation of fibroblasts and fibrogenesis in the kidney, which may be one of the molecular mechanisms associated with the progression of CKD [53].

Epigenetic patterns can change over one's lifetime, suggesting that epigenetic changes may constitute an important factor of the aging process [54]. Since CKD might be an aging-related disorder, we investigated the urine 5MedC level in different age categories in our CKD cohort. However, the CKD patients ≥ 75 years of age did not exhibit a significantly different level of urine 5MedC than those <75 years of age in our study (Table S2). We recognized the correlation between urine 5MedC, a marker of global DNA methylation, and urine 8-OHdG, a marker of oxidized DNA by oxidative stress (Figure 3), suggesting a link between DNA oxidation and DNA methylation. There might therefore be a connection between genetic and epigenetic alterations, possibly via oxidative stress in such patients. Several reports have investigated the relationship between oxidized DNA and DNA methylation [55–58], including the simultaneous examination of 8-OHdG and 5MedC in DNA samples [55]. 8-OHdG may induce hypomethylation of DNA by inhibiting DNA methylation at nearby cytosine bases [58]. Significantly negative correlations were reported between 8-OHdG and levels of global methylation in DNA extracted from leukocytes in workers exposed to nanomaterials of metal oxide [56] and between plasma 8-OHdG and global methylation levels in leukocyte DNA in subjects with biliary atresia [57]. Further investigations are thus required in order to clarify the association between oxidized DNA and DNA methylation.

This study has several limitations and strengths that must be kept in mind when understanding the data. First, urine 5MedC did not exhibit methylation of specific genes involved in CKD, such as *polycystic kidney disease 1* [59] but exhibited global DNA methylation. Second, while three major enzymes are necessary for de novo DNA methylation (DNMT3A and DNMT3B) or maintenance methylation (DNMT1) in mammalian cells [3], we did not examine the levels of these enzymes in the present study. Third, we did not have sufficient data on subjects with diabetic nephropathy, which is the most frequent cause of ESRD in several countries. However, including diabetic subjects in the CKD cohort may have influenced the 5MedC levels, as alterations in the DNA methylation of the gene network occur under conditions of diabetes mellitus [60] and in glomerular podocytes under conditions of diabetic nephropathy [61, 62]. Fourth, the serum and kidney tissue levels of 5MedC were not investigated in this study, as we did not obtain these samples in our setting. Renal compartment-specific genetic and epigenetic analyses would be able to identify further novel mechanisms involved in the progression of CKD [63]. Fifth, we were unable to evaluate cardiovascular events in CKD patients because we expected a low number of such events in our setting, although epigenetic dysregulation of CKD-associated cardiovascular disease might be relevant [64]. In addition, we were unable to confirm the level of 5MedC in our urine samples using other methodologies, such as LC-MS/MS [12]. Other caveats include the lack of data on lifestyle risks, such as smoking and toxin exposure, as potential confounders.

## 5. Conclusions

We examined the levels of urine 5MedC, a marker of DNA methylation and an epigenetic marker, in patients with CKD. These values significantly increased in the later CKD stages and were related to a reduced eGFR. The urine 5MedC level in combination with albuminuria or the *α*1MG level significantly predicted the renal survival in CKD patients, suggesting that it can serve as a novel biomarker for predicting the renal outcome in CKD, which is a significant issue given the currently increasing number of CKD patients all over the world. Our previous studies and others suggested urine trefoil factors to be biomarkers for progression of CKD [19, 65, 66]; however, recent studies demonstrated these small peptides as biomarkers for acute kidney injury [67] and drug-induced kidney injury [68]. We thus believe urine 5MedC as a promising and novel biomarker for CKD based on the current study.

Further studies to clarify the kidney disease-specific changes in the level of 5MedC utilizing a larger CKD cohort and exploring the renal compartment-specific epigenetic analyses will be necessary. Clarifying whether intervention and treatment of CKD patients with agents such as cholesterol-lowering medications [69] can alter the level of urine 5MedC is of great importance.

## Figures and Tables

**Table 1:** Baseline characteristics of the study subjects divided by CKD stages.

	All patients	Early to Mid-CKD (stages 1 to 3)	Later CKD (stages 4 and 5)	*P* value
N	308	241	67	
Age (years)	56 (37-67)	52 (35-65)	62 (55-71)	<0.0001
Gender, male, n (%)	164 (53.2)	123 (51.0)	41 (61.2)	0.139
eGFR (ml/min/1.73 m^2^)	55.4 (32.0-79.6)	63.8 (47.9-85.8)	18.6 (14.2-24.3)	<0.0001
UAE (mg/gCr)	158 (20-762)	89 (12-542)	705 (126-1431)	<0.0001
u*α*1MG (mg/gCr)	5.7 (2.1-14.1)	3.9 (1.7-8.5)	23.5 (11.8-48.7)	<0.0001
u5MedC (*μ*mol/gCr)	65.9 (40.8-130.3)	59.7 (39.0-116.5)	88.3 (48.5-153.9)	0.025
Hemoglobin (g/L)	130 (116-142)	133 (123-146)	112 (101-129)	<0.0001
MBP (mmHg)	91 (84-99)	91 (83-99)	96 (85-103)	0.013
Cause of CKD, n (%)				<0.0001
Chronic GN∗	157 (51.0)	146 (60.6)	11 (16.4)	
Nephrosclerosis	40 (13.0)	19 (7.9)	21 (31.3)	
Diabetic nephropathy	11 (3.6)	7 (2.9)	4 (6.0)	
Others∗∗	100 (32.5)	69 (28.6)	31 (46.3)	
Diabetes mellitus, n (%)	36 (11.7)	27 (11.2)	9 (13.4)	0.615
Current medication, n (%)				
ARBs/ACEIs	196 (63.0)	137 (56.9)	59 (88.1)	<0.0001
CCBs	117 (38.0)	72 (29.9)	45 (67.2)	<0.0001

Data are expressed as the median (interquartile) or number (percentage). *α*1MG, alpha1-microglobulin; ARB, angiotensin receptor blocker; ACEI, angiotensin-converting enzyme inhibitor; CCB, calcium channel blocker; CKD, chronic kidney disease; eGFR, estimated glomerular filtration rate; GN, glomerulonephritis; MBP, mean blood pressure; UAE, urinary albumin excretion; u*α*1-MG, urinary *α*1-microglobulin; u5MedC, urinary 5-methyl-2'-deoxycytidine. ∗Chronic glomerulonephritis includes 93 cases (59.2%) of IgA nephropathy, 22 cases (14.0%) of minimal change nephrotic syndrome, 12 cases (7.6%) of membranous nephropathy, 12 cases (7.6%) of IgA vasculitis with nephritis, 7 cases (4.5%) of focal segmental glomerulosclerosis, 6 cases (3.8%) of non-IgA mesangial nephritis, 4 cases (2.6%) of membranoproliferative glomerulonephritis and 1 case (0.6%) of acute glomerulonephritis (persistent and chronic phase). ∗∗Others include 62 cases (62.0%) of unknown etiology without a renal biopsy; 20 cases (20.0%) of lupus nephritis; 10 cases (10.0%) of anti-neutrophil cytoplasmic antibody-associated vasculitis; 3 cases (3.0%) of polycystic kidney disease; 2 cases (2.0%) of Alport syndrome; and 1 case each (1.0%) of thin basement membrane disease, cholesterol crystal embolization, and vesicoureteral reflux.

**Table 2:** A multiple logistic regression analysis to determine low eGFR (later CKD stages, <30 mL/min/1.73 m^2^) in different models.

	Odds ratio	95% CI	*P* value
Model 1			
u5MedC ≥ median (*μ*mol/gCr)	2.30	1.29 - 4.21	0.005
Model 2			
u5MedC ≥ median (*μ*mol/gCr)	2.16	1.18 – 4.04	0.012
UAE ≥ 300 (mg/gCr)	4.31	2.36 – 8.08	<0.0001
Model 3			
u5MedC ≥ median (*μ*mol/gCr)	2.36	1.24 - 4.60	0.008
UAE ≥ 300 (mg/gCr)	1.39	0.67 - 2.90	0.381
u*α*1MG ≥ median (mg/gCr)	13.56	5.32 – 40.1	<0.0001

Adjusted for age and gender. The median values of u5MedC and u*α*1MG are 65.9 (*μ*mol/gCr) and 5.7 (mg/gCr), respectively. CKD, chronic kidney disease; eGFR, estimated glomerular filtration rate; UAE, urinary albumin excretion; u*α*1-MG, urinary *α*1-microglobulin; u5MedC, urinary 5-methyl-2'-deoxycytidine; CI, confidence interval.

## Data Availability

No data is used to support this study.

## Supplementary Materials

Supplementary Materials Supplementary Table S1: univariate correlation between urinary 5MedC and other clinical parameters. Supplementary Table S2: urinary 5MedC levels according to age, gender, cause of CKD, and complication of diabetes. Supplementary Figure S1: the renal survival categorized by albuminuria, urine *α*1MG, and urine 5MedC alone.
