# Supplementary material for: Urine 5MedC, a Marker of DNA Methylation, in the Progression of Chronic Kidney Disease
Source: Dis Markers. 2019 Jul 1;2019:5432453. doi: 10.1155/2019/5432453 (PMC6636573; doi:10.1155/2019/5432453)
Supplement: Supplementary Materials — Supplementary Table S1: univariate correlation between urinary 5MedC and other clinical parameters. Supplementary Table S2: urinary 5MedC levels according to age, gender, cause of CKD, and complication of diabetes. Supplementary Figure S1: the renal survival categorized by albuminuria, urine α1MG, and urine 5MedC alone. [file 5432453.f1.pdf]

Urine 5MedC, a Marker of DNA Methylation, in the Progression of Chronic Kidney Disease

Akifumi Onishi, Hitoshi Sugiyama, Masashi Kitagawa, Toshio Yamanari, Keiko Tanaka, Ayu Ogawa-Akiyama, Yuzuki Kano, Koki Mise, Katsuyuki Tanabe, Hiroshi Morinaga, Masaru Kinomura, Haruhito A. Uchida, and Jun Wada

SUPPLEMENTARY INFORMATION

Table S1. Univariate correlation between urinary 5MedC and other clinical parameters

	R value	P value
Age (years)	-0.0140	0.806
eGFR (ml/min/1.73 m^2)	-0.0359	0.530
UAE (mg/gCr)	0.0419	0.464
uα1MG (mg/gCr)	0.0346	0.546
Hemoglobin (g/L)	0.0465	0.416
MBP (mmHg)	0.0752	0.188

eGFR, estimated glomerular filtration rate; 5MedC, 5-methyl-2’-deoxycytidine; MBP, mean blood pressure; UAE, urinary albumin excretion; uα1MG, urinary alpha1-microglobulin.

Table S2. Urinary 5MedC levels according to age, gender, cause of CKD and complication of diabetes

	Urinary 5MedC	P value
Age		
< 75 years	68.8 (41.2-130.6)	
≥ 75 years	49.6 (28.8-110.6)	0.119
Gender		
Male	64.7 (41.3-129.0)	
Female	70.9 (38.6-132.8)	0.732
Cause of CKD		
Glomerulonephritis	63.6 (41.7-112.6)	
Nephrosclerosis	70.2 (39.2-128.0)	
Diabetic nephropathy	105.7 (51.9-170.2)	
Others	64.8 (37.0-152.8)	0.515
Complication		
Diabetes mellitus	70.9 (39.1-123.4)	
No diabetes mellitus	65.1 (40.9-131.5)	0.943

Data (μmol/gCr) are expressed as the median (interquartile).

CKD, chronic kidney disease; 5MedC, 5-methyl-2'-deoxycytidine.

Figure S1

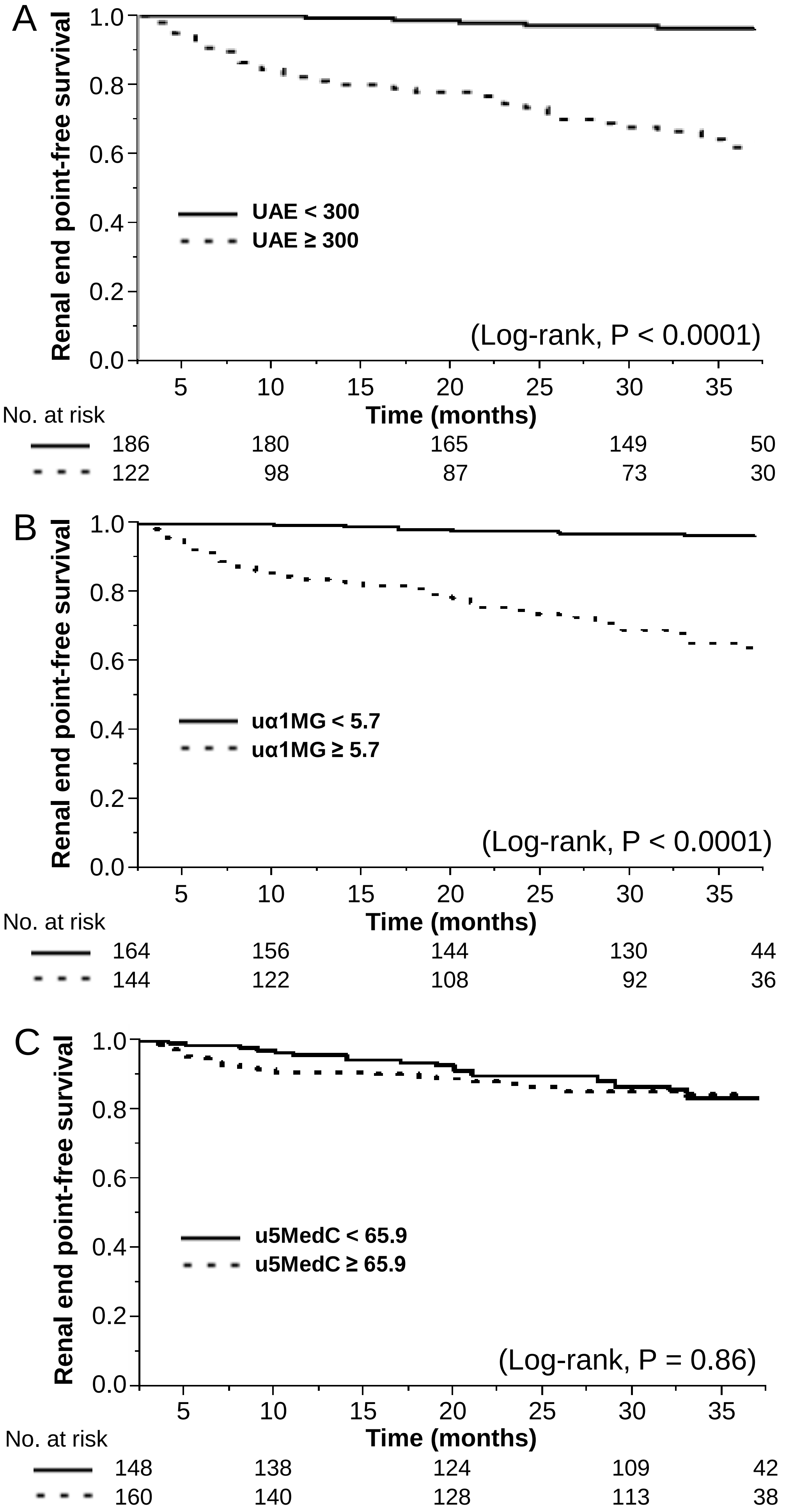
A
Renal end point-free survival
1.0
0.8
0.6
0.4
0.2
0.0
UAE < 300
UAE ≥ 300
(Log-rank, P < 0.0001)
5
10
15
20
25
30
35
Time (months)
No. at risk
186 180 165 149 50
122 98 87 73 30
B
Renal end point-free survival
1.0
0.8
0.6
0.4
0.2
0.0
uα1MG < 5.7
uα1MG ≥ 5.7
(Log-rank, P < 0.0001)
5
10
15
20
25
30
35
Time (months)
No. at risk
164 156 144 130 44
144 122 108 92 36
C
Renal end point-free survival
1.0
0.8
0.6
0.4
0.2
0.0
u5MedC < 65.9
u5MedC ≥ 65.9
(Log-rank, P = 0.86)
5
10
15
20
25
30
35
Time (months)
No. at risk
148 138 124 109 42
160 140 128 113 38

Supplementary Figure Legends

Fig. S1. The renal survival categorized by albuminuria, urine α1MG and urine 5MedC alone. Macroalbuminuria (mg/gCr) and the median value of urine α1MG (mg/gCr) predicted the three-year renal endpoint-free survival (A and B, respectively), while the median value of urine 5MedC (µmol/gCr) did not (C). Log-rank test. UAE, urinary albumin excretion; uα1MG, urinary alpha1-microglobulin; u5MedC, urinary 5’-methyl-2’-deoxycytidine.
